# Implications of Oxidative Stress in the Pathogenesis and Treatment of Hyperpigmentation Disorders

**DOI:** 10.1155/2022/7881717

**Published:** 2022-01-18

**Authors:** Xiaoxue Xing, Yanjun Dan, Zhongyi Xu, Leihong Xiang

**Affiliations:** Department of Dermatology, Huashan Hospital, Fudan University, Shanghai 200040, China

## Abstract

Oxidative stress represents an imbalance between the generation of reactive oxygen and nitrogen species and the ability of antioxidant systems to decompose those products. Oxidative stress is implicated in the pathogenesis of hyperpigmentation, hypopigmentation, melanoma, and other skin diseases. Regulatory networks involving oxidative stress and related pathways are widely represented in hypopigmentation diseases, particularly vitiligo. However, there is no complete review into the role of oxidative stress in the pathogenesis of hyperpigmentation disorders, especially regarding associations involving oxidative stress and cellular signaling pathways. Here, we review oxidative and antioxidant systems, oxidative stress-induced signal transduction mechanisms, and effects of antioxidant drugs used in preclinical and clinical settings in hyperpigmentation disorders.

## 1. Introduction

Hyperpigmentation disorders are diseases in which patches of skin are darker than the normal surrounding skin, resulting from the upregulated activity of melanin synthesis, increased numbers of melanocytes, and decreased decomposition of melanosomes [1]. Hyperpigmentation disorders include melasma, Riehl's melanosis, postinflammation hyperpigmentation (PIH), solar lentigo, and ashy dermatosis [2, 3]. Some primary hyperpigmentation conditions, such as nevocytic nevi, café au lait spots, may be inherited via autosomal-dominant genetics, which we will not discuss further in this paper [4]. The causes of hyperpigmentation include excessive sun exposure, exposure to a variety of topical products, exposure to cosmetics, pregnancy, inflammation, certain drugs, and some systemic diseases [5]. These are briefly listed in Table 1.

**Figure 1:**
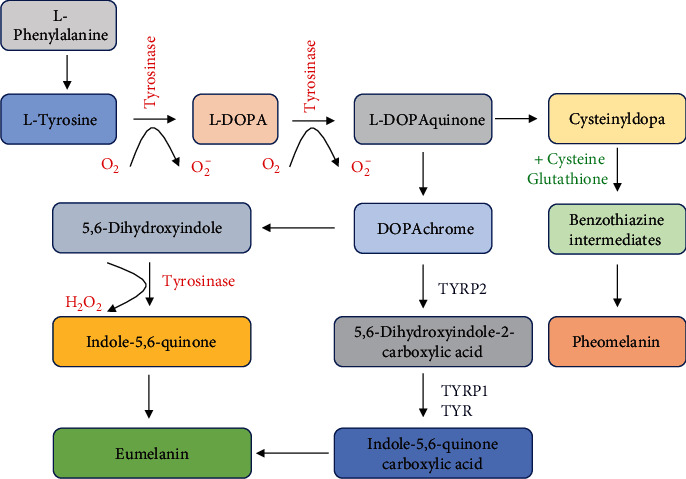
Tyrosinase oxidizes tyrosine to DOPA and DOPA to dopaquinone. The catalytic activity of tyrosinase results in the generation of O^2−^. Dopaquinone then is converted into dopachrome through a redox exchange. Dopachrome either generates dihydroxyindole (5,6-DHI), which is oxidized into indole quinone, or produces dihydroxyindole carboxylic acid (5,6-DHICA) by decarboxylation, and 5,6-DHICA is then converted into the corresponding quinone. Polymerization of these reactive quinones finally leads to the formation of the eumelanin. The synthesis of pheomelanin is involved in the generation of cysteinyl-DOPA; then, it is converted into benzothiazine derivatives.

Melanin synthesis by melanocytes involves oxidation reactions and generation of superoxide anions and hydrogen peroxide, which maintain melanocytes in a state of oxidative stress. Briefly, the generation of oxidative reactions includes the following: (1) Tyrosinase oxidizes L-tyrosine to L-3,4-dihydroxy-phenylalanine (L-DOPA) and L-DOPA to DOPAquinone. The catalytic activity of tyrosinase results in the generation of O_2_^−^. (2) Tyrosinase catalyzes 5,6-dihydroxyindole to indole-5,6-quinone, accompanied by generation of H_2_O_2_ [6], as depicted in Figure 1. Even though the oxidative species are generated, they are maintained at a certain level by a complex antioxidant system, paracrine factors, and gene regulatory networks to prevent cellular damages resulting from oxidative species. For example, endothelin-1 secreted by keratinocytes can reduce H_2_O_2_ generation and *α*-MSH to reduce oxidative DNA damage induced by H_2_O_2_ [7]. TRP2 inhibits oxidative stress by increasing glutathione levels and reducing DNA damage induced by free radicals [8]. It is reported that low concentrations of H_2_O_2_ can activate melanin synthesis and increase melanosome transfer into keratinocytes [9, 10]. It is well-established that UVA and visible light, which are risk factors for hyperpigmentation, stimulate pigmentation, oxidative stress, and proinflammatory cytokines.

In hyperpigmentation disorders, Seçkin et al. showed increased levels of malondialdehyde (MDA), nitric oxide (NO), and elevated enzymatic activity of superoxide dismutase (SOD) and glutathione peroxidase (GSH-Px) in the serum of melasma patients compared to controls [11]. It was also demonstrated that reactive oxygen species (ROS) such as nitric oxide are involved in the pathogenesis of PIH [12]. The above studies indicate that oxidative stress plays a pivotal role in the pathogenesis of hyperpigmentation disorders. It should be noted that ROS can readily react with DNA, lipids, or proteins and lead to cell death [13]. We thought the damage was eliminated by complex antioxidant systems and regulatory networks in hypermethylation disease. This process finally promotes melanocyte survival and melanin synthesis.

From the perspective of the role of oxidative stress in physiology and pathology, oxidative stress is tightly associated with autophagy, the mTOR and PI3K/Akt pathways, the P62/Keap1/Nrf2 system, and DNA damage and repair pathways [14]. The relationship between oxidative stress and the above signaling pathways has been studied in hypopigmentation diseases [15, 16]. However, in hyperpigmentation disorders, how the production of melanin is regulated under the stimulation of oxidative stress, and how the related pathways are involved in melanocyte survival and generation of melanin is not well understood. In this review, we discuss the activation of oxidative and antioxidant systems and associations involving oxidative stress and signal transduction pathways in hyperpigmentation disorders. We also summarize the use of antioxidant drugs in pre- or clinical settings.

## 2. Signaling Pathways

### 2.1. Oxidative and Antioxidant Systems

In response to signals from cytokines, infection, UV radiation, cosmetics, and drugs, melanocytes and keratinocytes produce ROS, including superoxide anion (O_2_^−^), peroxides, hydroxyl radicals (OH^−^), and singlet oxygen (O^2^) in the process of activating NADPH oxidases, the mitochondrial electron transport chain, nitric oxide synthases, and other enzymes [17]. Accordingly, cells have evolved antioxidant systems to scavenge ROS. These systems include enzymatic antioxidants such as SOD, catalase, glutathione peroxidase (GPX), and thioredoxin, as well as nonenzymatic antioxidants including vitamin C, vitamin E, coenzyme Q10, carotenoids, GSH (glutathione), ubiquinol, uric acid, melanin, and sulfhydryl [18, 19]. We describe these in Table 2.

In melasma, serum levels of MDA, SOD, and blood glutathione are significantly higher than those of controls. Choubey et al. also demonstrated that serum levels of MDA, NO, SOD, and GSH-Px are significantly higher in melasma patients than in controls [20]. Jo et al. found higher expression of inducible nitric oxide synthase (iNOS) and Akt phosphorylation in keratinocytes in skin biopsy specimens of melasma lesions [21]. In naevus and dysplastic naevus, the expression level of peroxiredoxin I, which is an enzymatic antioxidant, is increased [22], which indicates that oxidative stress and antioxidant systems are highly activated in hyperpigmentation disorders. Importantly, a number of studies have demonstrated that synthesis of melanin and survival of melanocytes are closely connected to oxidative stress. Hence, we hypothesized that a relevant signaling pathway should be also activated.

### 2.2. Wnt Signaling Pathway

Oxidative stress and the Wnt signaling pathway influence each other. Oxidative stress can dissociate interactions between nucleoredoxin (NXN, a thioredoxin-related redox-regulating protein) and disheveled (DVL, a key molecule in the Wnt signaling pathway), enabling DVL to interact with Frizzled (FZD, a transmembrane receptor). Subsequently, it induces *AXIN2* gene expression and nuclear accumulation of *β*-catenin and activates Wnt activity [23, 24]. Higher expression of Wnt1 in epidermal cells of melasma compared to normal control tissue has also been reported [25]. In lesioned skin of melasma patients, the expression of Wnts, both canonical and noncanonical, is increased. The expression of Wnt inhibitory factor-1 (*WIF-1*) is significantly reduced in melasma lesions compared to healthy normal control tissue. WIF-1 knockdown decreases glycogen synthase kinase-3b (GSK-3b), *β*-catenin, and NFATc2 (nuclear factor of activated T cells, cytoplasmic, calcineurin-dependent 2) phosphorylation and increases microphthalmia-associated transcription factor (*MITF*) expression [26]. It has also been demonstrated that activation of the canonical Wnt pathway positively influences melanogenesis. Cadherin 11 expression in fibroblasts and keratinocytes positively influences melanogenesis via the canonical Wnt and Akt activation pathways in cocultured melanocytes [27]. In solar lentigines, the expression of Wnt1, a ligand which activates the Wnt/*β*-catenin signaling pathway, is highly expressed through DNA hypomethylation and promotes the generation of melanin [28]. In summary, several studies have shown increased abundance of oxidative species in hyperpigmentation disorders, whereas the higher levels of oxidative species can activate Wnt signaling and promote melanin synthesis. However, we still do not know how oxidative stress balances the activation of the Wnt/*β*-catenin pathway. Whether the perturbed activation of the Wnt signaling pathway affects the state of oxidative stress also needs further investigation.

A number of studies indicate that the Wnt/*β*-catenin pathway is highly activated and induces melanin synthesis in hyperpigmentation disorders. Cardamonin, a chalcone from *Alpinia katsumadai* Hayata, was created to promote the degradation of intracellular *β*-catenin and inhibit pigmentation in melanocytes by suppressing the Wnt signaling pathway. It is a potential whitening agent for the treatment of hyperpigmentation disorders [29]. Additionally, the Wnt/*β*-catenin signaling inhibitor H89 can also reduce pigmentation [30]. However, its efficacy in treating hyperpigmentation disorders deserves further study.

### 2.3. Nrf2-ARE Pathway

The Nrf2- (nuclear erythroid 2-related factor 2-) ARE (antioxidant response element) pathway plays essential roles in initiating the expression of genes involved in cellular antioxidant and anti-inflammatory defenses, as well as mitochondrial protection [31]. Accumulating evidence shows that mitochondrial ROS (mtROS) activate Nrf2 [32]. In human melanocytes, the oxidative species and Nrf2-ARE pathway may also be closely associated. In response to oxidative stress or other exogenous insults, keratinocytes and melanocytes activate the expression of *Nrf2*, and then, the cells synthesize detoxification enzymes, such as *γ*-glutamylcysteine synthetase, heme oxygenase-1, and glutathione S-transferase, to mitigate the oxidative stress [33–35]. However, in solar lentigines and older photodamaged skin, the expression of Nrf2 and HO-1 is reduced [36]. We thought that the inhibition of the Nrf2-ARE pathway should favor melanin synthesis and protect the cell from apoptosis. In fact, the inhibitory effects of Nrf2 on melanin synthesis have been reported, as the overexpression of Nrf2 inhibits the expression of tyrosinase-related protein 1. However, the underlying mechanism remains unclear [37]. Importantly, some reports indicate that Nrf2 agonists, such as sulforaphane, marliolide, and its derivative, 5-methyl-3-tetradecylidene-dihydro-furan-2-one (DMF02), have the potential to be used in the treatment of hyperpigmentation disorders [36, 38], although additional clinical studies are required.

### 2.4. Autophagy

Research shows that autophagy is essential to maintain redox system homeostasis. ROS can activate the process of autophagy, which promotes cell adaptation and diminishes oxidative damage by degrading and recycling damaged intracellular macromolecules or dysfunctional organelles. Murase et al. reported that Caucasian skin exhibits higher autophagic activity than African American skin. Melanin levels in human skin cells cultured ex vivo and in human skin substitutes cultured *in vitro* were substantially diminished by activators of autophagy and enhanced by inhibitors [39]. It was also demonstrated by Kim et al. that the synthetic autophagy inducer PTPD-12 stimulates autophagic flux in human melanocytes and keratinocytes, and this increased autophagic flux leads to melanosome degradation without affecting the expression of MITF [40]. In addition, 585 nm light-emitting diodes (LED) suppress melanin content in human epidermal melanocytes by decreasing the expression of TRP-1 and MITF and inducing melanocyte autophagy, autophagosome accumulation, and LC3-II accumulation in melanocytes. These results suggest that 585 nm LED photomodulation has the potential to be used in the treatment of hyperpigmentation disorders [41]. It was also revealed that a potent agent named tranexamic acid used in treating melasma may function by activating the autophagy system in melanocytes [42]. The above studies indicate that autophagy induction promotes melanin degradation and could represent a target for treating hyperpigmentation disorders. Importantly, some scholars indicate that enhancement of ATG7-dependent autophagy protects melanocytes from oxidative stress-induced apoptosis [43]. Overall, these reports indicate that autophagy is finely controlled. How such balance is maintained remains unknown. We hypothesized that in this situation, autophagy should be activated to promote cell survival and melanin synthesis, while a higher level of autophagy would degrade the melanin.

Autophagy is a major sensor involved in redox signaling. Activation of autophagy is mainly mediated by the Nrf2 signaling pathway. When the autophagy flux is compromised, Nrf2 signaling is activated. Under oxidative stress, Nrf2 degradation is blocked, and the Nrf2-ARE pathway is also activated [44, 45]. In a hyperpigmentation model, Nrf2 induction and autophagy activation can be harnessed to increase melanosome degradation [34]. Kim et al. reported that arginase-2 upregulation reduces melanosome degradation by senescence-induced autophagy inhibition, resulting in hyperpigmentation in melasma [46]. In senile lentigo, a relationship between premature skin aging and diminished autophagy was also detected in hyperpigmented lesions of patients, while autophagy activation reduced pigmentation in ex vivo lesioned skin [47]. Those studies indicate that autophagy could be a target for regulating pigmentation. However, we still do not understand the exact genes that regulate autophagy signaling and affect the process of pigmentation in hyperpigmentation disorders.

### 2.5. DNA Damage and Repair

An association involving DNA damage and hyperpigmentation has been identified in nucleotide excision repair (NER) disorders. Patients with such disorders usually exhibit sensitivity to sun exposure and hyperpigmentation [48]. Mutation of RecQ protein-like 4 (RECQL4) causes Rothmund-Thomson syndrome [49], and the *STK11/LKB1* gene mutation results in dysfunction of UV-induced DNA damage responses and hyperpigmentation [50]. *In vivo*, oxidative DNA damage is an inevitable consequence of cellular metabolism. Among the nucleobases, guanine is the most susceptible to ROS attack because of its low redox potential. The main products of guanine oxidation are 8-hydroxyguanine and 8-hydroxydeoxyguanosine (8-OHG and 8-OHdG) [14]. Oxidized DNA base lesions are mainly removed by two types of pathways: base excision repair (BER), involving the removal of single lesions by a glycosylase action, and NER, which is a more complex process involving the removal of a lesion containing oligonucleotides [51]. Eller et al. demonstrated that DNA damage enhances melanogenesis. When human melanocytes are treated with the DNA-damaging chemical agent methyl methanesulfonate (MMS) or 4-nitroquinoline 1-oxide (4-NQO), cellular melanin content is increased by 70% and the mRNA level of tyrosinase is upregulated [52]. In atypical (dysplastic) nevi, there is greater melanin content and higher fragmentation of oxidized DNA than in normal melanocytes [9]. Importantly, oxidative DNA damage induces pigmentation and activates the NER pathway simultaneously, as shown by increased levels of XPA-binding protein 1 [53]. This indicates that DNA damage, DNA repair, and increased melanin synthesis are closely associated. However, the exact pathways and altered gene expression in DNA damage and DNA repair have not been explored in hyperpigmentation disorders.

### 2.6. Central Hub Role of P53

The activation of tumor suppressor p53 plays an essential role in controlling cell proliferation and death in response to oxidative stress and other factors [54, 55]. The Wnt signaling pathway, redox system, DNA repair, and PI3K-Akt pathway are connected via p53. It has been demonstrated that the process of hyperpigmentation is p53-dependent [56]. Casein kinase 1*α* (CK1*α*) is a critical regulator of Wnt signaling. Ablation of CK1*α* induces Wnt signaling and p53 activation. CK1*α* knockout in the keratinocytes is accompanied by *β*-catenin and p53 stabilization; the number of epidermal melanocytes and eumelanin levels are dramatically increased. *In vivo*, CK1*α* and P53 double-knockout mice fail to show epidermal hyperpigmentation, demonstrating that the process of hyperpigmentation is p53-dependent [56]. In periorbital hyperpigmentation, p53 single nucleotide polymorphisms (SNPs) are associated with different presentations of the disease [57]. It was also revealed that expression and phosphorylation of p53 are increased in the epidermal cells of hyperpigmented spots, accompanied by higher expression of melanogenic cytokines and significantly higher expression of p53 transcriptional targets [58]. In solar lentigines, the expression of p53 is increased, and it was found to be significantly correlated with acanthosis and cornification grade [59]. However, Espósito et al. compared the expression of p53 between facial melasma and normal adjacent skin and found no difference by calculating immunofluorescence intensity [25]. We thought that P53 activation may be varied in different disease types. Actually, a certain level of oxidative stress activates p53 expression and upregulates its antioxidant activities to eliminate ROS. However, higher levels of oxidative stress can lead to cell death [60]. Hence, we inferred that oxidative stress and tuned p53 activation in hyperpigmentation disorders favored melanocyte survival and melanin synthesis, while the exact roles of p53 and oxidative stress deserve further investigation.

### 2.7. Paracrine Regulation and Microenvironment

Melanocytes are known to be closely associated with keratinocytes and fibroblasts, as they secrete many factors that modulate the function of melanocytes, reduce oxidative damage, and maintain their survival.

In the epidermis, keratinocytes secrete many cytokines to regulate melanogenesis through paracrine effects. Studies have revealed that in terms of the expression of certain cytokines, including stem cell factor (SCF), endothelin 1 (ET-1) is significantly increased in hyperpigmented skin compared to normal control tissue. SCF is a physiological melanocyte growth factor that activates both the phosphatidyl-inositol-3 kinase (PI3K) and extracellular regulated kinase (ERK) pathways, which strongly protect melanocytes from apoptosis [58]. Endothelin 1 is an important keratinocyte-derived factor that regulates melanogenesis. The expression level of ET-1 in keratinocytes is increased upon exposure to exogenous insults, and ET-1 promotes the repair of oxidative DNA damage by inhibiting the generation of hydrogen peroxide [61, 62]. As mentioned above, keratinocytes secrete higher levels of *α*-MSH [41]. The antioxidant function of keratinocytes is exerted by increasing the activity of catalase and upregulating the expression level of heme oxygenase-1 [35]. Both *α*-MSH and ET-1 upregulate the expression of MCR1 and positively influence pigmentation [63].

Fibroblasts synthesize a number of cytokines regulating melanogenesis, such as fibroblast-derived basic fibroblast growth factor (FGF), SCF, hepatocyte growth factor (HGF), neuregulin-1, and TGF-beta [64, 65]. In melasma, fibroblasts derived from lesional and perilesional skin secrete more nerve growth factor- (NGF-) *β* than normal controls [66]. In fibroblasts of darker skin phototypes, the synthesis of a certain cytokine, neuregulin-1, which stimulates pigmentation, is increased [67]. These findings show that different types of paracrine factors play essential roles in regulating oxidative stress and hyperpigmentation.

In addition, immune cells participate in the process of hyperpigmentation. Skin wounding triggers a repair response that induces recruitment of neutrophils, melanoblasts, and melanocytes, which function to kill invading microbes and clear dead cells and matrix debris. These cells release cytokines such as stromal cell factor 1, directing the activities of other cells during the repair process and leading to wound hyperpigmentation [68]. However, future studies regarding the role of immune cells in hyperpigmentation disorders are needed.

## 3. Antioxidant Drugs

Great progress has been made in the treatment of hyperpigmentation disorders. Several novel agents have also gained attention for their potential skin-lightening abilities. The MMP2 inhibitor ARP101 induces autophagy and inhibits melanogenesis [69]. Gomisin N, a lignan compound found in *Schisandra chinensis*, inhibits melanin synthesis by repressing the expression of MITF and melanogenic enzymes [70]. Alpha-viniferin inhibits melanin production in melasma and freckles by inhibiting protein kinase A (PKA) activation and reassociation between catalytic and regulatory subunits in cAMP-elevated melanocytes [71]. Retinoids, retinol, silymarin, and flutamide show efficacy in the treatment of melasma and PIH [72]. In this section, we will highlight antioxidant drugs, while sparing the role of target inhibitors or other herbs in the treatment of hyperpigmentation.

### 3.1. Vitamin C

Vitamin C eliminates oxidative stress by the oxidation of ascorbate to monodehydroascorbate and then to dehydroascorbate. It is a cofactor for the enzymatic activity of prolyl hydroxylase during collagen synthesis. Vitamin C and its derivatives inhibit tyrosinase activity and melanin content in a dose-dependent manner [73]. Thus, it has been demonstrated to be a valuable and safe dermocosmetic depigmenting compound with a strong effect in preventing signs of photoaging and UV-induced hyperpigmentation [74]. In melasma and PIH, full-face iontophoresis of vitamin C appears to be an effective short-term treatment to improve the condition [75].

### 3.2. Azelaic Acid

A multicenter, randomized, double-blinded, parallel-group study measured the efficacy, safety, and tolerability of azelaic acid (20%) cream compared with vehicle only for the treatment of facial hyperpigmentation in dark-skinned patients. After a 24-week treatment period, azelaic acid produced significantly greater decreases in pigment intensity than the vehicle only [76]. Azelaic acid works against pigmentation by inhibiting melanin production and tyrosinase activity [77]. In addition, azelaic acid can reversibly inhibit respiratory chain enzyme activity, which includes NADH dehydrogenase, succinate dehydrogenase, and cytochrome C oxidoreductase. It also acts as a reversible inhibitor of thioredoxin reductase, cytochrome P450 reductase, and 5-alpha-reductase [78].

### 3.3. Vitamin E

Studies have demonstrated that topical and oral administration of different forms of vitamin E (including topical RRR-alpha-tocopherol (Eol), topical RRR-alpha-tocopheryl succinate, and oral RRR-alpha-tocopheryl acetate) can reduce inflammation and pigmentation [79]. The antioxidative mechanism of tocopherols partially results from the hydroxyl group in the chromanol ring, which donates a hydrogen atom to reduce free radicals. Alpha-tocopherol acetate suppresses IL-8 upregulation and expression of AP-1 through inhibition of NADPH oxidase activity and the formation of malondialdehyde-thiobarbituric acid. Its function of depigmentation is mediated by interfering with lipid peroxidation of melanocyte membranes, increasing intracellular glutathione content, and inhibiting tyrosinase activity [80]. While vitamin E alone shows minimal efficacy in the treatment of melasma [81], it is usually used with other antioxidant compounds.

### 3.4. Carotenoids

Carotenoids include *β*-carotene, astaxanthin, lutein, and zeaxanthin, etc. Carotenoids possess the ability to quench ^1^O_2_, protect against UV-induced ROS generation, and decrease antioxidant enzyme activities and membrane perturbation [82]. Topical application of beta-carotene lotion decreases melasma intensity index and lesion size [83]. A randomized, double-blinded, placebo-controlled clinical trial compared subjects with oral lutein/zeaxanthin treatment to a placebo group. The results revealed skin lightening effects within the treated cohort, which may be related to tyrosinase inhibition and increased antioxidant capacity [84]. However, oral lutein/zeaxanthin treatment had minimal effects in terms of PIH and long-term exposure to UV.

### 3.5. Polypodium leucotomos Extract (PLE)

The efficacy of *Polypodium leucotomos* extract (PLE) in reducing skin pigmentation is mainly mediated through quenching of free radicals, limiting membrane lipid peroxidation, and reducing superoxide anions, hydroxyl radicals, and singlet oxygen [85]. In the clinic, for melasma patients, the use of PLE could improve the melasma area severity index (MASI) scores, but the differences were not significant compared to the control group [86]. To date, no study has evaluated the effect of PLE on PIH. Considering the antioxidant function of PLE, it may have potential in treating PIH in the future.

### 3.6. Vitamin A

Vitamin A is the name of a group of fat-soluble retinoids, including retinol, retinal, and retinyl esters. Topical retinoids have been used to treat pigmentary disorders such as melasma, actinic lentigines, and PIH [87]. It is reported to be an effective tool for protecting mammary tissue against oxidative stress during lipopolysaccharide- (LPS-) induced acute mastitis in a rat model [88]. However, one study reported that patients receiving microneedling treatment with retinoic acid exhibited a significant reduction in SOD activity and a substantial increase in carbonyl levels, presenting as downregulation of nonenzymatic antioxidant defenses. The mechanism involving retinoids in regulating oxidative stress requires further investigation [89].

### 3.7. Lipoic Acid

In a three-dimensional human skin model, alpha-lipoic acid was reported to inhibit melanogenesis [90]. Alpha-lipoic acid shows great efficacy in improving UV-induced pigmentation and epidermal thickening in a mouse model [91]. Alpha-lipoic acid is a powerful antioxidant that can directly scavenge ROS and indirectly enhance the antioxidant defense network [92].

### 3.8. Glutathione

Glutathione is a thiol tripeptide that plays an essential role in maintaining redox balance. It directly or indirectly inhibits the activity of tyrosinase and switches the production of melanin from eumelanin to phaeomelanin [93]. An *in vivo* study showed that the level of glutathione in plasma had a strong negative correlation with the MASI score in patients with melasma [94]. An eight-week clinical study showed that patients treated with 500 mg of buccal glutathione lozenge had a significant reduction in melanin index [95], while patients treated with topical glutathione (2%) suspension also showed efficacy [96]. The use of glutathione is also recommended for treating patients with freckles and PIH.

### 3.9. Silymarin

Silymarin, an antioxidant drug, shows efficacy in melasma and improves clinical symptoms in a dose-dependent manner. Silymarin can scavenge ROS and enhance antioxidant enzymes and has been proven to be effective in the prevention of skin damage caused by UV [97, 98]. In an *in vitro* study, silymarin was considered to be a depigmenting agent, and its effects might be related to the inhibition of tyrosinase expression [99].

### 3.10. Cysteamine

In melasma patients, cysteamine cream showed significant efficacy in decreasing the melanin content of lesions in a randomized double-blind, placebo-controlled study [100]. In PIH, it also has a significant impact on reducing melanin production by inhibiting melanogenic enzymes in melanogenesis [101]. The function of cysteamine is mediated by providing cys and increasing the level of GSH in cells, which results in antioxidant activity [102].

### 3.11. Coenzyme Q10

Coenzyme CoQ10 (CoQ10) plays an important role in preventing lipid peroxidation and protecting tissues against oxidative damage. It is a ubiquinone compound that has been reported to inhibit tyrosinase activity and melanin production. CoQ10 downregulates melanin synthesis by suppressing MITF expression by suppressing cAMP-mediated CREB signaling cascades [103]. Its use in hyperpigmentation disorders is still under investigation.

### 3.12. Kojic Acid

Kojic acid acts as a ROS scavenger, exhibiting antioxidant properties and inhibiting tyrosinase [104]. One study compared the efficiency of a gel containing 10% glycolic acid and 2% hydroquinone with or without 2% kojic acid in patients with melasma. It was found that the use of gel containing kojic acid resulted in greater improvement in melasma (60% of cases) compared with the gel without kojic acid (47.5% of cases) [105]. It was also demonstrated by Deo et al. that kojic acid in synergy with hydroquinone can significantly improve MASI scores compared with those obtained using other combinations [106].

### 3.13. Selenium

A number of studies have demonstrated the important role of selenium in antioxidant selenoproteins for protection against ROS [107]. Selenium has been used as a nutritional supplement and medicine because of its potential antioxidant effects at low concentrations. Selenium-containing carbohydrates can inhibit melanin synthesis [108]. Mice treated with selenium and vitamin E exhibit significantly less acute and chronic UV-induced skin damage [109]. Until now, there have been no clinical studies to measure the function of selenium specifically in hyperpigmentation disorders, as most studies have only investigated the antipigment role of selenium-containing compounds combined with other antioxidant drugs.

### 3.14. Niacinamide

Niacinamide, which is also called nicotinamide, is one of two major forms of vitamin B3. Supplementation with nicotinamide restores cellular NAD^+^ pools and mitochondrial energetics, attenuates oxidative stress and inflammatory responses, enhances extracellular matrix and skin barrier, and inhibits the pigmentation processes [110]. In clinical studies, niacinamide significantly improved hyperpigmentation and increased skin lightness compared with vehicle alone after 4 weeks of use [111]. A double-blinded, randomized clinical trial of topical 4% niacinamide versus 4% hydroquinone in the treatment of melasma indicated that niacinamide is a safe and effective therapeutic agent by decreasing pigmentation, inflammatory infiltration, and solar elastosis [112].

## 4. Conclusions

**Figure 2:**
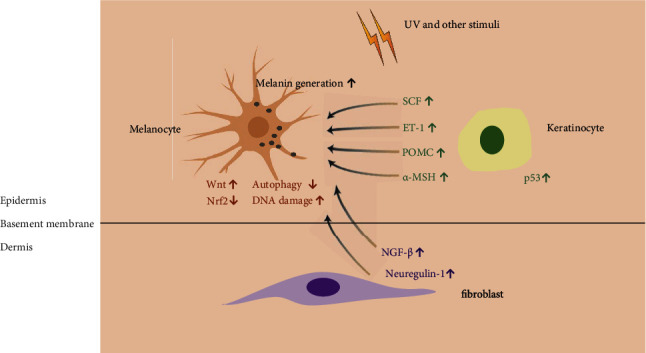
Role of oxidative stress in hyperpigmentation disorders.

Accumulating evidence shows that the activation of signaling pathways by oxidative stress is mediated by modifications of DNA, lipids, and proteins. Covalent modifications involving thiol groups of cysteine residues of KEAP1 initiate the activation of the Nrf2-ARE pathway [113]. The formation of MDA and 4-HNE results from the modification of lipid by free radicals; these products are associated with PI3K/Akt and NF-KB signaling pathways [114]. Importantly, oxidized lipid species activate cellular antioxidant systems and are detoxified by glutathione and glutathione peroxidase 4, which are related to the ferroptosis pathway [115]. DNA damage caused by oxidative stress activates DNA repair signaling [116]. All of these pathways play essential roles in the formation of melanin and pathogenesis in hyperpigmentation disorders, which are demonstrated by the number of *in vivo* and ex vivo studies. We depict some of these in Figure 2.

Under oxidative stress (such as UV and other stimuli), P53 was activated in keratinocytes, and keratinocytes synthesized higher levels of SCF, ET-1, POMC, and *α*-MSH to promote melanin generation. Fibroblasts also synthesized and secreted more NGF-*β* and neuregulin-1 to have paracrine regulation on melanogenesis. Meanwhile, oxidative stress could cause the induction of the Wnt signaling pathway and DNA damage and the downregulation of the Nrf2 pathway and autophagy.

Several studies have shown the depigmentary capacity of antioxidant drugs, whereas single therapeutics may not result in very significant efficacy. A single oral dose of N-acetylcysteine (NAC) does not effectively protect nevi from UV-induced oxidative stress [117]. Vitamin E alone showed minimal efficacy in the treatment of melasma [79]. Combinations of different antioxidant drugs are being investigated to treat hyperpigmentation. A compound containing vitamin C, vitamin E, and other extracts exerts antiaging and brightening effects on the skin [118, 119]. Plant herbs have been shown to have a multitude of cellular effects in various dermatological diseases [120]. Apart from that, a number of small-molecule agonists or inhibitors are now being investigated in preclinical trials to treat hyperpigmentation disorders. Therefore, targeting signaling pathways may represent a very promising therapeutic approach for improving hyperpigmentation conditions.

## Figures and Tables

**Table 1:** Causes of hyperpigmentation.

(i) External factors
Excess sun exposure, UV
Exposure to moderate heat or infrared radiation
Cosmetic products
Topical products
Constant scratching
Allergy
Chemotherapy drugs
Injury and inflammation
Infection
(ii) Internal factors
Pregnancy
Hormonal causes
Aging
(iii) Certain diseases
Diabetic dermopathy
Addison disease
Systemic lupus erythematosus
Hyperthyroidism
Acanthosis nigricans
Hereditary hemochromatosis
Postchikungunya fever pigmentation
Lichen planus
Actinic lichen planus

**Table 2:** Oxidative and antioxidant systems involved in pigmentation.

(i) Endogenous sources of ROS
NADPH oxidases
Mitochondrial electron transport chain
Nitric oxide synthases
Lipoxygenases
Xanthine oxidases
Cyclooxygenases
Cytochrome P450 enzymes
Polyamine and amino acid oxidases
Endoplasmic reticulum
Peroxisomes
(ii) Enzymatic antioxidant
Superoxide dismutase
Catalase
Glutathione peroxidase
Glutathione S-transferase
Glutathione reductase
Thioredoxin
Peroxiredoxins
(iii) Nonenzymatic antioxidant
Vitamin C
Vitamin A
Vitamin E
Coenzyme Q10
Glutathione
Ubiquinone
Uric acid
Melanin
Melatonin
Selenium
Carotenoids
Flavonoids
